# Detection of *Gnathostoma spinigerum* larva in the brain with complete follow-up after surgical treatment of human neurognathostomiasis

**DOI:** 10.1016/j.fawpar.2024.e00229

**Published:** 2024-05-11

**Authors:** Chayanuchit Chayangsu, Sumate Ampawong, Onrapak Reamtong, Parnpen Viriyavejakul, Tapanee Kanjanapruthipong, Kamonpan Fongsodsri, Suwaphat Intapun, Pongwat Polpong, Rattanarat Intarat, Prakaykaew Charunwatthana, Abigail Hui En Chan, Dorn Watthanakulpanich

**Affiliations:** a Navavej International Hospital, Bangkok, Thailand 10230; b Department of Tropical Pathology, Faculty of Tropical Medicine, Mahidol University, Bangkok, Thailand 10400; c Department of Molecular Tropical Medicine and Genetics, Faculty of Tropical Medicine, Mahidol University, Bangkok, Thailand 10400; d Neurosurgery Department, Neurological Institute of Thailand, Bangkok, Thailand 10400; e Department of Surgery, Nopparat Ratchathani Hospital, Bangkok, 10230, Thailand; f Department of Clinical Tropical Medicine, Faculty of Tropical Medicine, Mahidol University, Bangkok, Thailand 10400; g Department of Helminthology, Faculty of Tropical Medicine, Mahidol University, Bangkok 10400, Thailand

**Keywords:** Human neurognathostomiasis, Craniotomy, evidently detected, Proteomic analysis

## Abstract

Human gnathostomiasis is a food-borne zoonotic helminthic infection widely reported in Latin America, Asia, and Southeast Asia. Consuming raw, or under-cooked fresh-water fish is the leading cause of this helminthic infection, which is clinically characterized by signs of inflammation, itching sensation, or irritation with migratory swelling. Neurological symptoms resulting from neurognathostomiasis vary, and there is scant information due to the rareness of patient brain samples. This study aimed to demonstrate the first evidence of human neurognathostomiasis by the detection of *Gnathostoma spinigerum* larva in patient's brain during craniotomy, supported by histopathological, immunological and proteomic evidence. Clinical symptoms were obtained from medical history and physical examination with laboratory investigations, including magnetic resonance imaging (MRI), left temporal craniotomy, histopathology of brain tissue, and Western blot analysis, were performed to elucidate the causative pathogens for diagnosis. In addition, the host–parasite interaction of the parasite invading the patient's brain was characterized through proteomics. Histopathology revealed worms with the characteristic cuticular spines of *G. spinigerum* which were detected and identified. These histopathological findings were consistent with a positive Western blot showing a 24-kDa reactive-band for gnathostomiasis. Proteomic analysis revealed the presence of *G. spinigerum* serpin and serine protease in the patient's serum. Moreover, the leucine-rich alpha-2-glycoprotein was indicated as a systemic biomarker of early brain injury related to invasion by *G. spinigerum.* Therefore, our study provides the initial evidence of human neurognathostomiasis due to *G. spinigerum* larval invasion along with successful craniotomy and proven larval detection including complete follow-up, and the disease prognosis after surgical treatment.

## Graphical abstract

**Figure:**
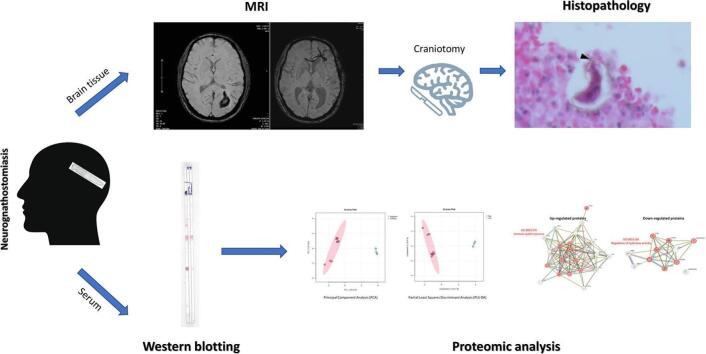

## 1 Introduction

Human gnathostomiasis is endemic in Thailand, where infection occurs due to the consumption of raw or under-cooked fresh-water fish containing *G*. *spinigerum* larva. As humans are accidental hosts, *G. spinigerium* is unable to develop into mature adult worms, resulting in larval migration to various tissues and organs (Daengsvang, 1980). The most common clinical manifestation of gnathostomiasis is localized-intermittent subcutaneous migratory swelling associated with localized piercing pain, pruritus, and erythema. These symptoms usually last for 1–2 weeks and may persist resulting from the mechanical disruption of tissues due to larval migration (Daengsvang, 1981; Dekumyoy and Watthanakulpanich, 2019). Erratic migration of *Gnathostoma* larvae to the skin, subcutaneous tissues, liver, heart, lungs, kidneys, and brain has been reported in humans (Miyazaki, 1991; Sharma et al., 2017; Sivakorn et al., 2020). Tissue swelling results from inflammation due to tissue damage from larval migration (Daengsvang, 1981). Moreover, neurognathostomiasis may result from the invasion of the central nervous system (CNS), including the brain, and the spinal cord, and the critical signs and symptoms include radiculomyelitis or radiculomyeloencephalitis, eosinophilic meningitis or meningoencephalitis, subarachnoid or intracerebral hemorrhages, and ultimately death, as evidenced by CNS damage (Punyagupta et al., 1990; Katchanov et al., 2011). Death is highly probable, particularly with damaging to vital structures in the brainstem following the onset of CNS symptoms, as observed in postmortem cases, or if the larva burrows through a cerebral arteriole, resulting in massive subarachnoid hemorrhages (Chitanondh and Rosen, 1967; Punyagupta et al., 1968; Boongird et al., 1977). For neurognathostomiasis cases, the presence of even a single worm can be life-threatening. In Thailand, 6% and 18% of subarachnoid hemorrhages in adults and children, respectively, are attributed to gnathostomiasis (Visudhiphan et al., 1980).

Clinical manifestations, pathogenesis, and disease severity dependent on interactions between the host and parasite. The parasite possesses determinants of virulence, allowing it to successfully invade, damage, and resist host defenses. Conversely, the host also exhibits various degrees of resistance and defense mechanisms. As there is limited information available due to sporadic cases and the challenges involved in larval removal from the brain (Lee et al., 1988), this study focused on exploring the host–parasite interaction and the proteins involved in *Gnathostoma* larval invasion in the human brain. To the best of our knowledge, no study has investigated the disease prognosis of a patient's post-craniotomy due to neurognathostomiasis. Therefore, this study will contribute to our understanding of the changes occurring following larval migration and removal.

## 2 Materials and methods

### 2.1 Ethics approval

This study was approved by the Human Research Ethics Committee of the Faculty of Tropical Medicine, Mahidol University, Bangkok, Thailand (MUTM 2022–062-01).

### 2.2 Clinical details

A 36-year-old man presented with acute severe headache and loss of right lateral vision two days prior admission. He was healthy with no underlying diseases before the sudden onset of symptoms. Upon medical examination, right incongruous homonymous hemianopia was detected, with no meningeal abnormalities observed, and his neurological examination was normal. Initially, conservative treatment was administered; intravenous Tramadol was given as needed to control the headache. However, on the second day of admission, his clinical symptoms worsened, with altered consciousness and rhythmic jerking movements of the right index finger indicative of a seizure. The seizure was observed and controlled by intravenous Diazepam (10 mg), intravenous Dilantin (300 mg/day), and intravenous Levetiracetam (2000 mg/day).

### 2.3 Magnetic resonance imaging (MRI)

MRI was conducted in conjunction with basic laboratory investigations, including a complete blood count (CBC), urinalysis, fasting blood sugar, liver function test, and erythrocyte sedimentation rate (ESR) to determine the cause of the patient's symptoms. The MRI brain 3.0 Tesla Sagittal T1W, Axial T1W, T2W, FLAIR, SWI, DWI and ADC mapping Coronal GRE T2W, FLAIR, Post contrast T1W in sagittal, axial, and coronal planes was conducted following to the manufacturer's guidelines (Philips, Netherland). The MRI acquisition comprised a T1-weighted Magnetization Prepared 2 Rapid Acquisition Gradient Echo (MP2RAGE), a T2-weighted Turbo Spin Echo (TSE), and a diffusion MRI. The MP2RAGE sequence provided improved gray–white contrast at a high field compared to the classic MPRAGE acquisition. Subsequently, the brain images were interpreted by a radiologist.

### 2.4 Craniotomy

A craniotomy was performed on June 23, 2022, to excise the suspected causative pathogen and repair the traumatic injury site. Specifically, a left temporal craniotomy was performed, involving abscess removal and resection of the middle and inferior temporal lobes. Following the induction of general anesthesia, the patient was placed in a supine position and the patient's skull was fixed to a Mayfield clamp. The surgical site was meticulously prepared using sterile techniques. Subsequently, an incision was made in the skin, allowing access for the left temporal craniotomy. The abscess and affected portions of the middle, and inferior temporal lobes were identified and removed. Hemorrhaging bleeding was controlled through cauterization, and the dura matter was closed using 3–0 silk sutures. The skin and subcutaneous tissues were closed by standard techniques.

### 2.5 Histopathology

The brain tissue obtained was immersed in 10% formalin for 24 h to facilitate fixation and prepared for histopathological analysis. Subsequently, the processed brain tissue underwent standard evaluation using Hematoxylin and Eosin staining techniques.

### 2.6 Western blot analysis

Antibodies against suspected helminths (*G. spinigerum*, *Angiostrongylus cantonensis*, and *Toxocara* spp.) based on the initial differential diagnosis of the patient, were detected in the serum using an in-house confirmatory Western blot protocol, following established protocols as described elsewhere (Nopparatana et al., 1991; Dekumyoy et al., 2000)).

### 2.7 Proteomic study

A study on host–parasite interaction in brain tissue with larval migration was conducted through proteomic analysis. The patient's serum and three healthy sera were used. Serum proteins were precipitated with ice-cold acetone (1:5 *v*/v). After precipitation, the protein pellet was reconstituted in 15 mM ammonium bicarbonate (Sigma Aldrich Co., Missouri, United States) in 0.25% RapidGest SF (Waters Co., Massachusetts, United States). The whole protein (30 μg) was subjected to sulfhydryl bond reduction using 5 mM Dithiothreitol (Sigma Aldrich Co., Missouri, United States) in 15 mM ammonium bicarbonate for 1 h at 72 °C. The sulfhydryl alkylation was carried out using iodoacetamide (Sigma Aldrich Co., Missouri, United States) in 15 mM ammonium bicarbonate at room temperature for 30 min in the dark. The resulting solution was cleaned using a Zeba Spin Desalting Column (Thermo Scientific Co., Massachusetts, United States). Trypsin (Promega Co., Madison, United States) was used at 1:50 (enzyme–protein ratio) for digestion and incubated for 24 h at 37 °C. The digested solution was dried and reconstituted with 0.1% formic acid before being submitted to LC–MS/MS. Three technical replicates were performed for each sample.

An HF-X Hybrid Quadrupole-OrbitrapTM Mass Spectrometer and an EASY-nLC1000 nanoLC system outfitted with a nano C18 column were used in this experiment. The LC–MS/MS spectrum data were acquired in the positive mode. Mobile phase A had 0.1% formic acid in water, while mobile phase B contained 90% acetonitrile with 0.1% formic acid. Over the gradient of 135 min at a constant flow rate of 300 nL/min, 5% mobile phase A was used to re-equilibrate the column. The separation was carried out using a linear gradient of 2% to 45% mobile phase B, and 90% mobile phase B was used for 10 min to regenerate the analytical column. A data-dependent collection approach was used to examine the peptides.

MaxQuant v2.4.2.0 software was used to analyze the raw mass spectra (.raw file) and identify the proteins using the *G. spinigerum* NCBI and Human Uniprot databases. The following parameters were used for protein identification and quantification: 20 ppm MS tolerance, 0.05 Da MS/MS tolerance, trypsin as the digestion enzyme, cysteine carbamidomethylation as a fixed modification, and methionine oxidation as a variable modification. The false discovery rate was set to 1% to identify peptides and proteins. The relative abundance of proteins was normalized using the software's normalization algorithm (total intensity count). The proteins with fold change levels of ≥2 and *p*-value ≤0.05 were identified as differential expressions. The STRING database was used to analyze protein interactions. The principal component analysis (PCA) and partial least squares–discriminant analysis (PLS–DA) were analyzed by MetaboAnalyst 5.0 software.

## 3 Results

### 3.1 Clinical symptoms

The patient presented with acute severe headache accompanied by loss of right lateral vision. Additionally, the patient showed right incongruous homonymous hemianopia, alteration of consciousness, rhythmic jerking movement of the right index finger, and confusion. At present, the patient's condition has improved with no headache.

### 3.2 MRI scan findings

#### 3.2.1 Before the definite diagnosis

**Fig. 1:**
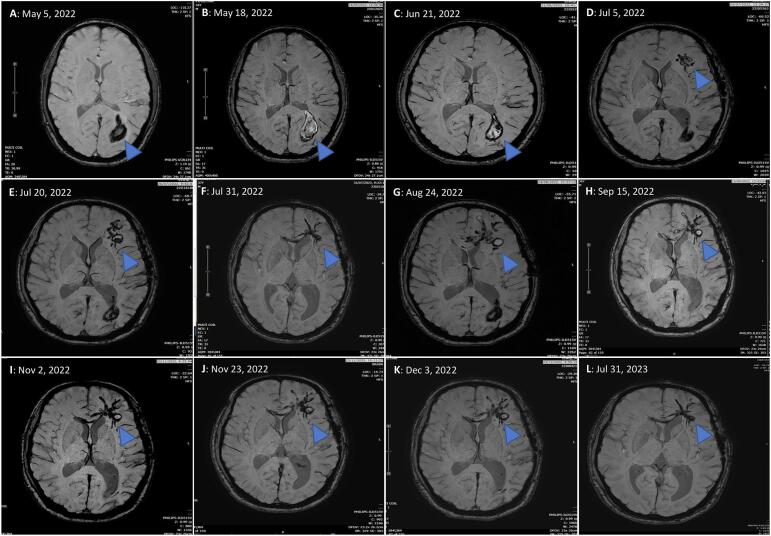
(A-L) MRI images of the patient's brain in series: Fig. A showed intraparenchymal hemorrhage in the left parietooccipital lobe (arrowhead) with mild perilesional edema; Figs. B, C showed a reduction in the size of occipital lobe hematoma two weeks pre-craniotomy (arrowheads), along with a new rim-enhancement lesion with a more invasive serpiginous hemorrhagic tract in the left temporal lobe; Fig. D showed brain edema in the left temporal lobe (arrowhead) following the definite diagnosis, the treatment with albendazole along with intravenous dexamethasone was administered, then treatment was stopped and ivermectin was administered alternatively, Figs. E showed progression of serpiginous hemorrhagic lesions, Figs. F-J showed stable serpiginous hemorrhagic lesions during combined treatment using albendazole and ivermectin for full four courses subsequently, Fig. K showed serpiginous hemorrhagic tract ceased, indicating the cessation of *Gnathostoma* larval worm migration, Fig. L showed no new serpiginous hemorrhagic tracts and improved necrotic lesions in the patient's brain.

An MRI was performed to investigate the suspected cause of symptoms. The MRI images (Figs. 1A-L) revealed interesting and significant findings. The initial image on May 05, 2022, displayed intraparenchymal hemorrhage in the left parietooccipital lobe measuring 4.1 × 2.0 × 4.8 cm in greatest diameter (arrowhead), accompanied by mild perilesional edema (Fig. 1A). Subsequently MRI scans, conducted two weeks pre-craniotomy on May 18, 2022, showed slight progression of the lesion (arrowhead), and on June 21, 2022, depicted a reduction in the size of occipital lobe hematoma with minimal perilesional edema, along with the identification of a new rim-enhancement lesion with a more invasive serpiginous hemorrhagic tract in the left temporal lobe (Figs. 1B, C). The definite diagnosis of neurognathostomiasis was determined on July 5, 2023.

#### 3.2.2 After the definite diagnosis

Following the definite diagnosis, the treatment was initiated with albendazole (200 mg, 2 tablets twice a day) along with intravenous dexamethasone for three days. However, treatment was abruptly discontinued due to impaired liver function test results. Consequently, ivermectin (200 μg/kg/day) was administered for two days as an alternative. However, brain edema was detected in the left temporal lobe, coupled with increased headache and confusion experienced by the patient (Fig. 1D). Single treatment seemed unsuccessful, as evidenced by the progression of serpiginous hemorrhagic lesions observed in the MRI on July 20, 2022, accompanied by persistent headache (Fig. 1E).Subsequently, a full combined treatment using albendazole (21 days) and ivermectin (2 days) was initially prescribed on July 20 to August 10, with the continuation of the combined treatment for three additional courses subsequently administered to the patient on August 24 to September 15, October 5 to October 26 and November 2 to November 23, 2022, respectively (Figs. 1F-J). The patient's condition exhibited improvement, and the progression of serpiginous hemorrhagic tract ceased, indicating the cessation of *Gnathostoma* larval worm migration (Fig. 1K). Further MRI scans revealed no new hemorrhagic tract and improved necrotic lesions with no progression in the patient's brain upon completion of the medication courses (Fig. 1L).

### 3.3 Craniological and histological findings

**Fig. 2:**
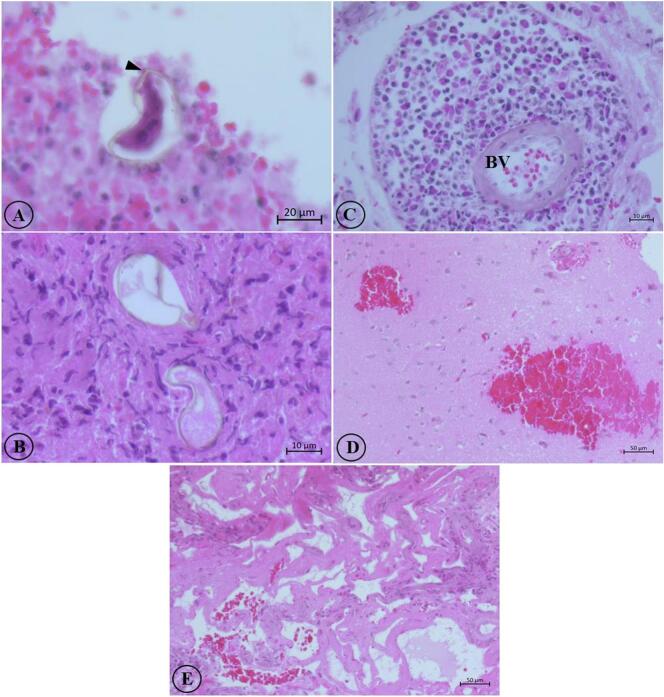
Histopathological findings of suspected gnathostomiasis invading brain. A-B show fragments of larva within the brain tissue, possible cuticles are discernible (arrowhead); C-E display histopathological findings of perivascular eosinophilic infiltration (C), petechial hemorrhages (D), and capillary proliferation (E). BV- blood vessel.

A multiloculated abscess containing 5 ml of frank pus was discovered within the left temporal lobe. Fragile tissue was observed within the early encapsulated capsule. The parasite itself was not visualized during surgery. The brain biopsy specimen from the left temporal lobe consisted of multiple pieces of soft brown tissues, collectively measuring 2.0 × 2.0 × 0.5 cm^3^. The section showed fragments of brain tissue with multifocal areas of acute and chronic inflammation. Deeper sections revealed small, round to oval parasites exhibiting undulating cuticular spines within the necrotic areas and leptomeninges (Figs. 2A-B). Numerous eosinophils were scattered in the brain tissues and predominantly concentrated around blood vessels (Fig. 2C). In addition, areas of petechial bleeding in the brain and capillary proliferation were present (Figs. 2D-E). The brain biopsy results highly suggested the presence of *Gnathostoma* worm within the brain.

### 3.4 Laboratory results

**Fig. 3:**
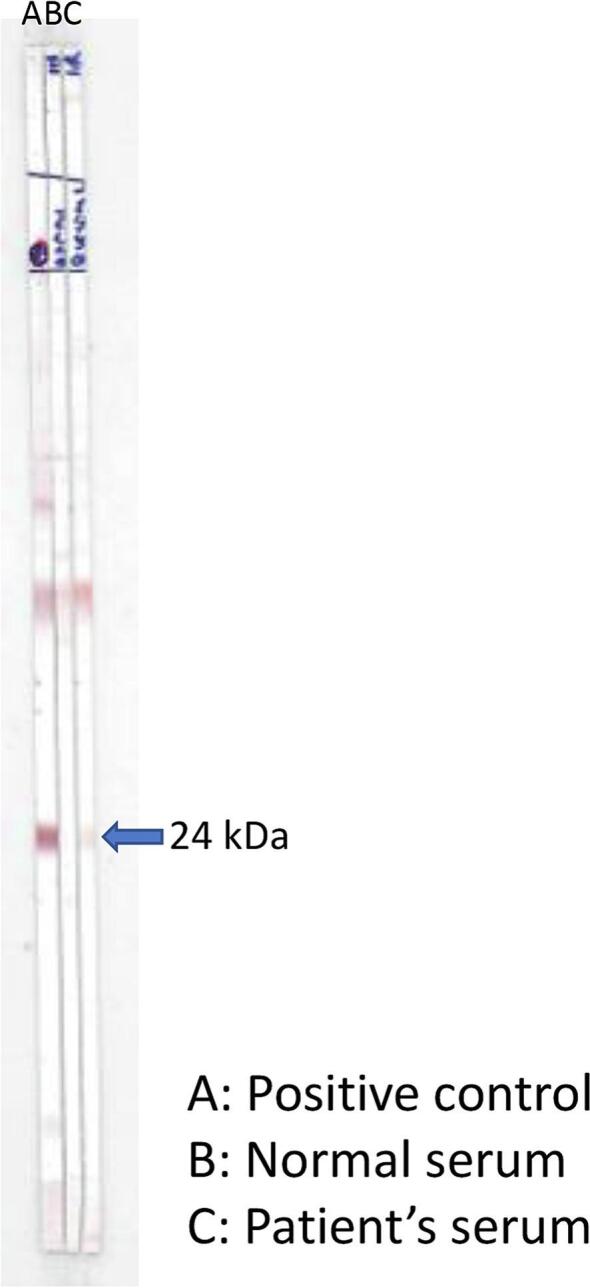
Western blot results of the patient's serum showing a positive 24 kDa reactive-band. Lanes A, B, and C indicate positive control, uninfected serum, and the patient's serum, respectively.

The patient's blood examination on June 25, 2022, revealed a total white blood cell count of 9990/mm^3^, with differentials showing 54 neutrophils, 23 lymphocytes, 4 monocytes, 1 basophil, and 18 eosinophils. The other parameters appeared normal. The CBC analysis demonstrated eosinophilia in peripheral blood, with an absolute eosinophil count of 1798 cells/mm^3^. Collaborating with the histopathological results, a positive reactive-band at the 24 kDa on Western blot analysis and elevated blood eosinophilia levels confirmed the diagnosis of gnathostomiasis (Fig. 3). Additional laboratory tests, such as the C-reactive protein test, showed abnormally high results at 16.2 mg/l (above the normal limit of <5 mg/l), indicating tissue damage and brain inflammation. The ESR was also elevated at 33.0 mm/h attributed to increased protein in the bloodstream (normal range: 0–15.0 mm/h) which is indicative of the severity of inflammation in certain brain tissues.

**Fig. 4:**
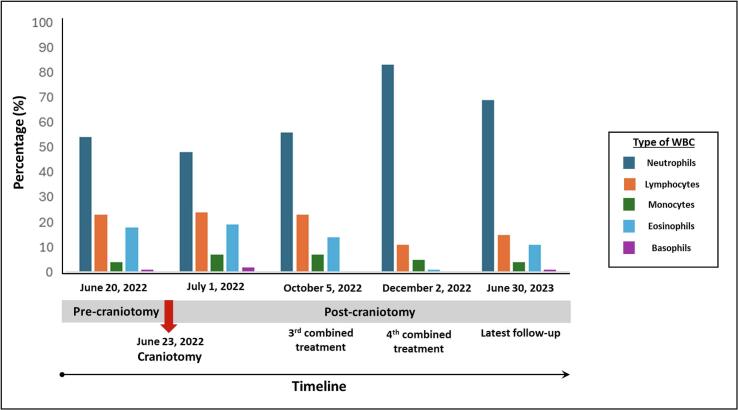
Hemogram of the patient's blood focusing on white blood cells distribution, especially eosinophils in various periods of neurognathostomiasis.

The hemogram monitoring (Fig. 4) post-craniotomy revealed persistent high eosinophils at 19%, corresponding with MRI findings of a new rim-enhancement lesion with an invasive serpiginous hemorrhagic tract in the left temporal lobe. However, there was a subsequent decrease in eosinophils to 14%, coinciding with the third full combined treatment for another larval migration. Following the fourth full combined treatment and confirmation of larval migration cessation by MRI, eosinophils decreased to 1% on December 2, 2022. The latest follow-up on July 30, 2023, indicated a slight increase in eosinophils to 11% which is hovering near the borderline fluctuation.

### 3.5 Proteomic results

**Fig. 5:**
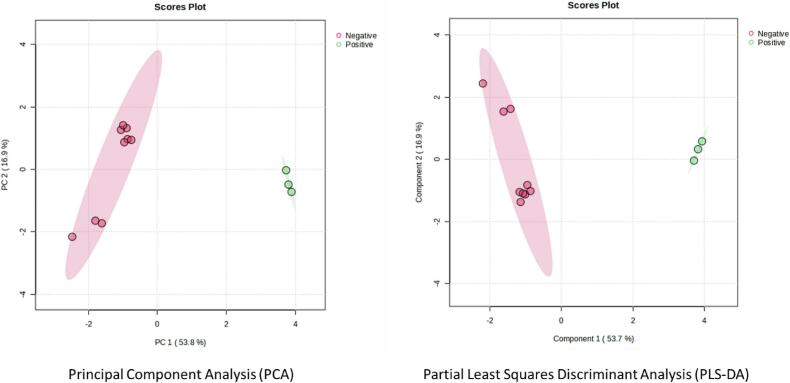
Principal component analysis (PCA) and partial least squares–discriminant analysis (PLS-DA) of human proteins between *G. spinigerum* uninfected and infected sera. Red and green represent the data from the negative (healthy serum) and positive (infected sera), respectively. (For interpretation of the references to colour in this figure legend, the reader is referred to the web version of this article.)

**Fig. 6:**
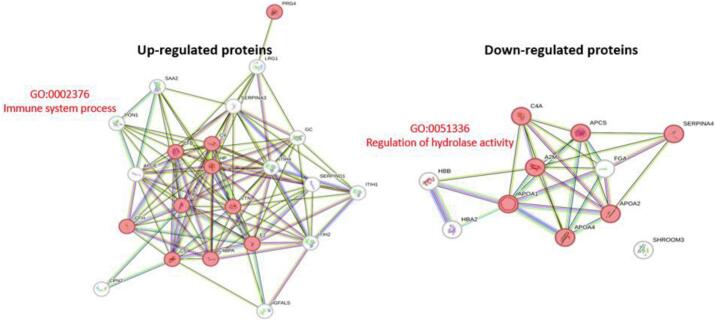
Protein-protein interactions of up-regulated and down-regulated neurognathostomiasis human serum proteins. The significant gene ontologies were identified as enriched and highlighted in the red line. The immune system process showed up-regulation, while the regulation of hydrolase activity was down-regulated. (For interpretation of the references to colour in this figure legend, the reader is referred to the web version of this article.)

Two circulating proteins from *G. spinigerum* were identified, where serpin and serine protease could be identified with scores of 25 and 22, respectively (Table 1). Additionally, twenty-three up-regulated and thirteen down-regulated human serum proteins were also observed (Table 2, Table 3). Among them, the serum amyloid A-2 protein, leucine-rich alpha-2-glycoprotein, and complement C5 were the most up-regulated, while the immunoglobulin heavy constant alpha 1, kallistatin, and complement C4-A were the most down-regulated. The PCA and partial least squares–discriminant analysis (PLS–DA) further supported the difference in human protein expression between uninfected and infected sera (Fig. 5). Using the STRING database to analyze protein–protein interactions, the immune system process was the dominant gene ontology for up-regulated proteins, while the regulation of hydrolase activity was identified as the central gene ontology of the down-regulated proteins (Fig. 6).

**Table 1:** Circulating proteins of *G. spinigerum* identified in the neurognathostomiasis patient's serum.

No	Accession no.	Protein	Score	M.W.	%coverage	pI
1	QEE82882.1	serpin, partial [Gnathostoma spinigerum]	25	39,918	15.6	6.28
2	ACA30304.1	serine protease [Gnathostoma spinigerum]	22	37,999	8.2	9.33

**Table 2:** Up-regulated human serum proteins from the neurognathostomiasis patient.

Protein	Score	Ratio	ANOVA
P0DJI9|SAA2_HUMAN Serum amyloid A-2 protein	16.199	44.06959	6.03E-07
P02750|A2GL_HUMAN Leucine-rich alpha-2-glycoprotein	13.559	23.854	0.010787
P01031|CO5_HUMAN Complement C5	23.488	10.10807	0.00093
P08603|CFAH_HUMAN Complement factor H	8.4329	8.420249	0.001061
P00734|THRB_HUMAN Prothrombin	13.676	8.167057	0.001148
P02748|CO9_HUMAN Complement component C9	13.901	7.727313	5.23E-07
Q92954|PRG4_HUMAN Proteoglycan 4	5.3566	6.502228	9.36E-05
P00751|CFAB_HUMAN Complement factor B	22.346	6.238373	0.00076
P35858|ALS_HUMAN Insulin-like growth factor-binding protein complex acid labile subunit	11.448	6.160359	0.004536
P04003|C4BPA_HUMAN C4b-binding protein alpha chain	9.8739	5.926734	0.006411
P22792|CPN2_HUMAN Carboxypeptidase N subunit 2	5.5415	3.899523	2.62E-05
P01024|CO3_HUMAN Complement C3	323.31	3.617335	2.02E-08
P04004|VTNC_HUMAN Vitronectin	6.0974	3.138488	6.62E-08
P27169|PON1_HUMAN Serum paraoxonase/arylesterase 1	4.5683	2.746723	0.349424
P00738|HPT_HUMAN Haptoglobin	139.11	2.680741	2.03E-05
P01011|AACT_HUMAN Alpha-1-antichymotrypsin	96.494	2.670199	2.78E-05
P19827|ITIH1_HUMAN Inter-alpha-trypsin inhibitor heavy chain H1	42.28	2.631917	4.74E-06
P02774|VTDB_HUMAN Vitamin D-binding protein	50.825	2.378416	4.73E-08
P01861|IGHG4_HUMAN Immunoglobulin heavy constant gamma 4	49.101	2.351452	0.000486
Q14624|ITIH4_HUMAN Inter-alpha-trypsin inhibitor heavy chain H4	99.44	2.343006	3.12E-08
P02649|APOE_HUMAN Apolipoprotein E	42.413	2.147796	0.001836
P05546|HEP2_HUMAN Heparin cofactor 2	18.948	2.021618	0.000584
P19823|ITIH2_HUMAN Inter-alpha-trypsin inhibitor heavy chain H2	55.213	2.01924	5.86E-05

**Table 3:** Down-regulated human serum proteins from the neurognathostomiasis patient.

Protein	Score	Ratio	ANOVA
P01876|IGHA1_HUMAN Immunoglobulin heavy constant alpha 1	86.466	0.49631	0.001947
P29622|KAIN_HUMAN Kallistatin	4.9156	0.49544	0.00615
P0C0L4|CO4A_HUMAN Complement C4-A	5.0808	0.494747	0.110555
P01023|A2MG_HUMAN Alpha-2-macroglobulin	323.31	0.345528	0.000761
P02652|APOA2_HUMAN Apolipoprotein A-II	22.532	0.325449	0.000105
P01859|IGHG2_HUMAN Immunoglobulin heavy constant gamma 2	38.733	0.31055	0.000652
P02647|APOA1_HUMAN Apolipoprotein A-I	193.63	0.298622	0.00021
P06727|APOA4_HUMAN Apolipoprotein A-IV	80.334	0.258186	0.007782
Q8TF72|SHRM3_HUMAN Protein Shroom3	4.1818	0.253339	0.294966
P02671|FIBA_HUMAN Fibrinogen alpha chain	20.373	0.22049	4.45E-05
P68871|HBB_HUMAN Hemoglobin subunit beta	37.728	0.135954	0.000325
P69905|HBA_HUMAN Hemoglobin subunit alpha	15.704	0.128111	0.000402
P02743|SAMP_HUMAN Serum amyloid P-component	20.465	0.062639	7.26E-07

## 4 Discussion

The patient had severe headaches and loss of right lateral vision due to mechanical trauma caused by the larval worm migration into the brain, indicative of neurognathostomiasis, which may present as a stroke-like syndrome, presenting with sudden bilateral occipital headaches (Smith et al., 2021). Additionally, the patient exhibited symptoms including alteration of consciousness, rhythmic jerking movement of the right index finger, and confusion. Daengsvang (1949) previously reported a case characterized by symptoms of meningeal irritation, especially headache, intermittent convulsions, and semiconsciousness lasting for about seven days, indicating the presence of the worm in the intracranium, similar to our patient's symptoms of depressed consciousness. However, our patient did not progress to a coma or mental confusion (Boongird et al., 1977; Punyagupta et al., 1990).

Confirmation of neurognathostomiasis was facilitated by a combination of MRI, craniological and histological analysis, and other laboratory tests including serology and proteomics. Initially, medical imaging techniques such as MRI and computerized tomography (CT) can assist in the presumptive diagnosis of human gnathostomiasis in cases where larvae have not been recovered (Kanpittaya et al., 2012; Chotmongkol et al., 2015). However, imaging techniques are nonspecific and nonconfirmatory, and they may not exclude other infections that can cause CNS symptoms. High-resolution MRI outperforms CT in the neuroimaging of cerebral larva migrans caused by *Gnathostoma* spp. as it can image the characteristic serpiginous *Gnathostoma* L3 worm tract, significantly increasing the accuracy of neurognathostomiasis diagnosis (Sawanyawisuth et al., 2004; Smith et al., 2021). The hallmark signs of gnathostomiasis include hemorrhagic tracts throughout the spinal cord and cerebral tissue postmortem, which were also presented in the patient in this study (Boongird et al., 1977; Chandenier et al., 2001; Suksathien et al., 2016).

Second, the presence of worm sections with the cuticular spines in the brain tissue obtained after surgery and histological analysis, alongside observed mechanical brain damage, suggests neurognathostomiasis. Direct mechanical damage can occur as the larva may burrow through a cerebral arteriole, leading to subarachnoid hemorrhages (Lo Re 3rd and Gluckman, 2003; Sithinamsuwan and Chairangsaris, 2005). The migratory pathway was likely through the loose connective tissues of the neural foramina of the skull base and the intervertebral foramina along nerve roots and vessels directly to the CNS. Larval migration can cause parenchymal damage, and subarachnoid hemorrhages (Katchanov and Nawa, 2010). Moreover, due to the tiny size of *Gnathostoma* larval worm, it may have been missed during surgery.

Third, laboratory analysis, including a confirmatory Western blot test and high eosinophilia, supported the diagnosis of neurognathostomiasis and ruled out other helminthic infections causing eosinophilic meningitis. Initially, the patient was diagnosed with possible parasitic eosinophilic meningoencephalitis, given his history of raw meat consumption and other tissue helminths, such as *Angiostrongylus* or *Toxocara* larval worms, were also considered as primary differential diagnoses due to similar symptoms. (Lo Re 3rd and Gluckman, 2003; Smith et al., 2021). However, neurognathostomiasis became highly probable when the brain images were revealed subarachnoid or intracranial hemorrhage with high eosinophils (Pirompakdi, 1984). In addition, the *Gnathostoma* larva is more invasive than *Angiostrongylus* larva, often leading to more frequent focal neurological signs (Lo Re 3rd and Gluckman, 2003; Lucas et al., 2008). Specific signs like acute nerve root pain, signs of spinal cord compression, and hemorrhagic or xanthochromic spinal fluid, differentiated neurognathostomiasis from *Angiostrongylus* infection. (Punyagupta et al., 1990; Lo Re 3rd and Gluckman, 2003).

Lastly, proteomics analysis revealed the presence of *G. spinigerum* serpin and serine proteases in the patient's serum. Previous studies found that the excretory–secretory products of the third-stage *G. spinigerum* larvae include serpins, and it has been demonstrated that these serpins react with sera from gnathostomiasis patients (Nuamtanong et al., 2019; Nogrado et al., 2022). By reducing host protease activity and modifying host immunity, parasite protease inhibitors thus act to make a safer environment for the host (Ranasinghe and McManus, 2017). Regarding serine proteases, several have been found in parasitic helminths that may contribute to immune evasion, host tissue and cell invasion, parasite growth and nutrition, and anticoagulation (Thiangtrongjit et al., 2021). Consistent with the damage and invasion observed in the brain due to *G. spinigerum*, the leucine-rich alpha-2-glycoprotein was the second most up-regulated protein. This protein has been reported as a systemic biomarker of early brain injury (Miao et al., 2022). Based on the protein-protein interaction analysis, the immune system process exhibited up-regulation, likely indicating a host immune response following parasite infection. Conversely, the regulation of hydrolase activity was observed to be down-regulated. In general, hydrolases are enzymes that catalyze bond cleavage through hydrolysis, primarily functioning in digestion to break down nutrients into smaller units. However, the specific roles of hydrolases in parasitic diseases remain poorly understood.

Neurological symptoms of neurognathostomiasis vary depending on the extent of larval migration, often leading to complications and a poor prognosis due to destruction of brain tissues and hemorrhages (Miyazaki, 1991; Kulkarni et al., 2015). Multiple hemorrhagic lesions can worsen the prognosis. (Wongpaiboonwatana and Nilaratanakul, 2020). Innitially, our patient improved with post-craniotomy and anthelminthic therapy, but worsening symptoms of progressive headache with slight confusion and elevated liver enzymes (increased direct bilirubin 0.38 mg/dl) on day 10th day of treatment possibly indicated increased cerebral hemorrhage. Albendazole was then discontinued, and intravenous dexamethasone (4 mg) every 6 h was administered to prevent the CNS reaction and relieve focal neurologic deficits (Punyagupta et al., 1990). In neuronathostomiasis, corticosteroids may play an additional role in reducing and suppressing CNS inflammation, and intensive neurologic care is essential to minimize brain edema in critical cases (Dekumyoy and Watthanakulpanich, 2019). Some cases may respond well to corticosteroids alone, without anthelminthic treatment (Smith et al., 2021). For our patient, ivermectin (0.2 μg/kg) was administered as an alternative medication instead of albendazole for two consecutive days, following recommendations for treating gnathostomiasis (Nontasut et al., 2005; Kanjanapruthipong et al., 2022). Albendazole and ivermectin monotherapy or in combination have been effective with initial experienced treatment failure (Chappuis et al., 2001; Parola et al., 2004). After completing four combined treatments with albendazole and ivermectin, the parasite ceased movement, and the patient's condition gradually improved with no headache. The only residual deficits were mild naming deficits, slight reduction in the right visual field of both eyes, and recent memory problem resulting from the partial removal of the inferior temporal lobe. Additionally, the patient's right homonymous hemianopia was resolved, and he was seizure-free with Levitiracetam 2000 mg/day treatment. The latest MRI showed no significant progression of the serpiginous hemorrhagic tract (Fig. 1L). Post-treatment follow-up, including monitoring antibody levels and eosinophilia is helpful as symptoms may reappear after a quiescent period (Dekumyoy and Watthanakulpanich, 2019). Supportive care with paracetamol significantly relieved the headache, while a decrease in eosinophils suggests treatment efficacy as demonstrated in Fig. 4. Life-threatening complications can arise from CNS parasitosis (Katchanov and Nawa, 2010), emphasizing the need for careful monitoring and follow-up. A practical criterion for determining cure is the absence of symptoms for more than one year, along with improvements in eosinophilia and antibody levels (Kraivichian et al., 2004). A follow-up of 6–12 months is recommended, as a long silent interval could precede a sudden recurrence of symptoms (Strady et al., 2009; Dekumyoy and Watthanakulpanich, 2019).

## 5 Conclusions

We present a case of neurognathostomiasis caused by *G. spinigerum* and provide the evidences of detecting *G. spinigerum* larvae following a successful craniotomy and MRI showing serpiginous hemorrhagic tracts. The diagnosis, prognosis, treatment, and follow-up measures were also successfully conducted. Therefore, we recommend that clinicians caring for patients with neurognathostomiasis consider using MRI to aid diagnosis, with confirmation of the diagnosis by a combination of methods including serology, histopathology and proteomics.

## CRediT authorship contribution statement

**Chayanuchit Chayangsu:** Resources, Investigation, Formal analysis, Data curation, Writing – review & editing, Writing – original draft. **Sumate Ampawong:** Investigation, Formal analysis, Writing – review & editing. **Onrapak Reamtong:** Investigation, Formal analysis, Writing – review & editing, Writing – original draft. **Parnpen Viriyavejakul:** Investigation, Formal analysis, Data curation, Writing – review & editing, Writing – original draft. **Tapanee Kanjanapruthipong:** Investigation, Formal analysis. **Kamonpan Fongsodsri:** Investigation, Formal analysis. **Suwaphat Intapun:** Resources, Investigation, Formal analysis, Data curation, Writing – original draft. **Pongwat Polpong:** Resources, Investigation, Formal analysis, Data curation, Writing – original draft. **Rattanarat Intarat:** Resources, Investigation, Formal analysis, Data curation, Writing – original draft. **Prakaykaew Charunwatthana:** Resources, Investigation, Formal analysis, Data curation. **Abigail Hui En Chan:** Writing – original draft. **Dorn Watthanakulpanich:** Visualization, Validation, Supervision, Resources, Project administration, Investigation, Formal analysis, Data curation, Conceptualization, Writing – review & editing, Writing – original draft.

## Declaration of competing interest

The authors declare that they have no known competing financial interests or personal relationships that could have appeared to influence the work reported in this paper.
